# Trends in robotic upper gastrointestinal and hepatopancreatobiliary surgery in Australia: a private sector-based analysis

**DOI:** 10.1007/s11701-026-03524-0

**Published:** 2026-06-01

**Authors:** Edwin Hur-Thompson, Sameesh Gupta, Sarah Duffy, Xiyu Chen, Alice Richardson, Ngee-Soon Lau, Christos Apostolou

**Affiliations:** 1 School of Medicine and Psychology, Australian National University, Canberra, Australia; 2 Sydney Adventist Hospital Clinical School, Australian National University, Sydney, Australia; 3 Statistical Support Network, Australian National University, Canberra, Australia; 4 Department of HPB and Upper GI Surgery, Canberra Hospital, Canberra, Australia; 5 Department of Surgery, Sydney Adventist Hospital, Sydney, Australia; 6 Clark Tower, Level 2 Suite 220, 185 Fox Valley Road, Wahroonga, NSW 2076 Australia

**Keywords:** Robotic surgical procedures, Pancreatectomy, Pancreaticoduodenectomy, Cholecystectomy

## Abstract

Robotic surgery is increasingly adopted across surgical specialties because of advantages in visualisation, dexterity, and ergonomics. However, data on its use in upper gastrointestinal (UGI) and hepatopancreatobiliary (HPB) surgery in Australia remain limited. This study characterises trends in robotic UGI and HPB surgery in Australia using data from the da Vinci System and Medicare Benefits Schedule (MBS) Item Reports website. Robotic procedure counts between 2013 and 2023 were obtained from Device Technologies Australia, local distributor of the da Vinci System by Intuitive Surgical in Australia. Corresponding MBS item numbers were used to determine UGI and HPB procedure volumes. Descriptive statistics, Poisson regression, and linear models were used to analyse trends over time. Robotic UGI and HPB surgery volume increased on average by 33% annually (95% CI 31%, 35%) over the study period, adjusted for surgery type. The proportion of MBS-claimed UGI and HPB procedures performed robotically increased on average by 0.16% (95% CI 0.12%, 0.20%) and 0.11% (95% CI 0.07%, 0.15%) per year, respectively. Cholecystectomy was the most common HPB procedure performed robotically but accounted for only 0.6% of MBS-claimed procedures. A considerable proportion of MBS-claimed left-sided pancreatectomies (34.4%) and pancreatoduodenectomies (18.1%) were performed robotically in 2023. Robotic bariatric procedures were the most commonly performed UGI procedure, although procedure counts plateaued after 2022. Robotic surgery for UGI and HPB procedures increased significantly over the study period. Notably, there has been a recent rise in the adoption of robotic approaches for complex non-bariatric procedures, particularly pancreatic surgery.

**Supplementary Information:**

The online version contains supplementary material available at 10.1007/s11701-026-03524-0.

## Introduction

Robotic surgery is steadily emerging as a mainstay of minimally invasive approaches, with the technology addressing many of the limitations of conventional laparoscopy [1, 2]. Since its introduction in Australia in the early 2000s, robotic surgery has been increasingly adopted by hospitals in both the public and private sectors, particularly in urology, gynaecology, and general surgery [3, 4]. Of these specialties, urology has led in surgical volume [4], while general surgery has seen the largest growth in recent years, primarily driven by robotic colorectal surgery and, more recently, hernia surgery [3].

Robotic surgery offers both technical advantages and potential improvements in patient outcomes [5]. From a technical perspective, it provides surgeons with enhanced visualisation, depth perception, dexterity, and ergonomics [5]. Patients may also have reduced complications, postoperative pain, and length of hospital stay [6–8]. Upper gastrointestinal (UGI) and hepatopancreatobiliary (HPB) surgery is often technically complex, requiring dissection of deep anatomical structures, vascular control, and intricate reconstruction in confined operative spaces [9–12]. These pose significant challenges for conventional minimally invasive approaches, which are often limited by the rigidity of laparoscopic instruments and the constraints of two-dimensional visualisation [9, 10]. The introduction of robotic surgical systems offers a potential solution to these limitations, making minimally invasive UGI and HPB operations more feasible [13].

In Australia, previous studies have characterised the trends in both urology and colorectal surgery [4, 14]. In urology, significant uptake of robotic surgery means that robotic approaches for procedures such as prostatectomy and partial nephrectomy have become the standard of care in the private sector [4]. Similarly, there has been an overall increase in the percentage of colorectal procedures performed robotically, with restorative rectal resections, rectopexies and right hemicolectomies accounting for a large proportion of procedures [14]. Conversely, while the adoption of robotic UGI and HPB surgery globally has been slower than in other specialties [15, 16], there is no published data specifically characterising its use in the Australian setting.

We use data from the Medicare Benefits Schedule (MBS) to characterise trends in robotic UGI and HPB surgery in Australia. Healthcare in Australia is delivered through a combination of public and private services. Private-sector care is funded through both government reimbursement via Medicare, Australia’s universal health insurance scheme and private health insurance or out-of-pocket contributions. MBS data capture government-reimbursed activity in the private sector but do not include procedures performed in public hospitals or funded outside the Medicare system. It thus serves as a useful proxy for private-sector activity in Australia.

Understanding how robotic approaches are being adopted for UGI and HPB surgery is key to guiding the development and evaluation of guidelines, surgical training, and allocation of funding in Australia [17, 18]. Furthermore, characterising the volume and type of robotic cases performed may also help identify the level of establishment of existing Australian programs.

## Aims

This study aims to characterise the utilisation of robotic surgery for UGI and HPB procedures in Australia from 2013 to 2023 by comparing robotic case volumes with MBS claim data.

### Methods

The methods used in this study were adapted from those previously published by Larach et al. [14]. Data on the number of general surgery robotic procedures performed between January 2013 and December 2023 in Australia were obtained from Device Technologies Australia, the local distributor of the da Vinci Robotic System (Intuitive Surgical, Sunnyvale, CA, USA). The data were provided as annual calendar year procedure counts. Comparator procedure volumes were extracted from the MBS Item Reports website [19] using procedure-specific item numbers corresponding to each procedure type. The MBS Item Reports website provides publicly available data on services billed to Medicare by eligible providers.

UGI procedures analysed included oesophagectomy, gastrectomy, hiatal hernia repair, oesophagogastric myotomy (OGM), gastric bypass, and sleeve gastrectomy. Robotic oesophagectomy included transcervical and transhiatal non-transthoracic procedures as well as chest and neck anastomoses made via transthoracic approaches. The number of oesophagectomies billed was obtained via the following MBS item numbers, 30,750, 30,751, 30,753, 30,754. Gastrectomy procedures, including distal, proximal, subtotal, and total resections, were identified using item numbers 30,518, 30,521, 30,526, and 30,762. Hiatal hernia repairs, including approaches for both paraoesophageal and sliding types, were identified using 31,468, 30,530, 31,466, and 30,529. OGM was captured using item numbers 30,532 and 30,533. Gastric bypass and sleeve gastrectomy were identified using item numbers 31,571 and 31,575, respectively.

The HPB procedures analysed were pancreatectomy, pancreatoduodenectomy, liver resection and cholecystectomy. Pancreatectomy, including distal and segmental resections, was identified using item numbers 30,583 and 30,792. Pancreaticoduodenectomy was identified using item number 30,584. Liver resections, including both formal anatomical and wedge resections, were identified using item numbers 30,414, 30,415, 30,418, and 30,421. Cholecystectomy procedures were identified using item numbers 30,443, 30,445, 30,448, 30,449, and 30,455.

### Statistical analysis

**Fig. 1:**
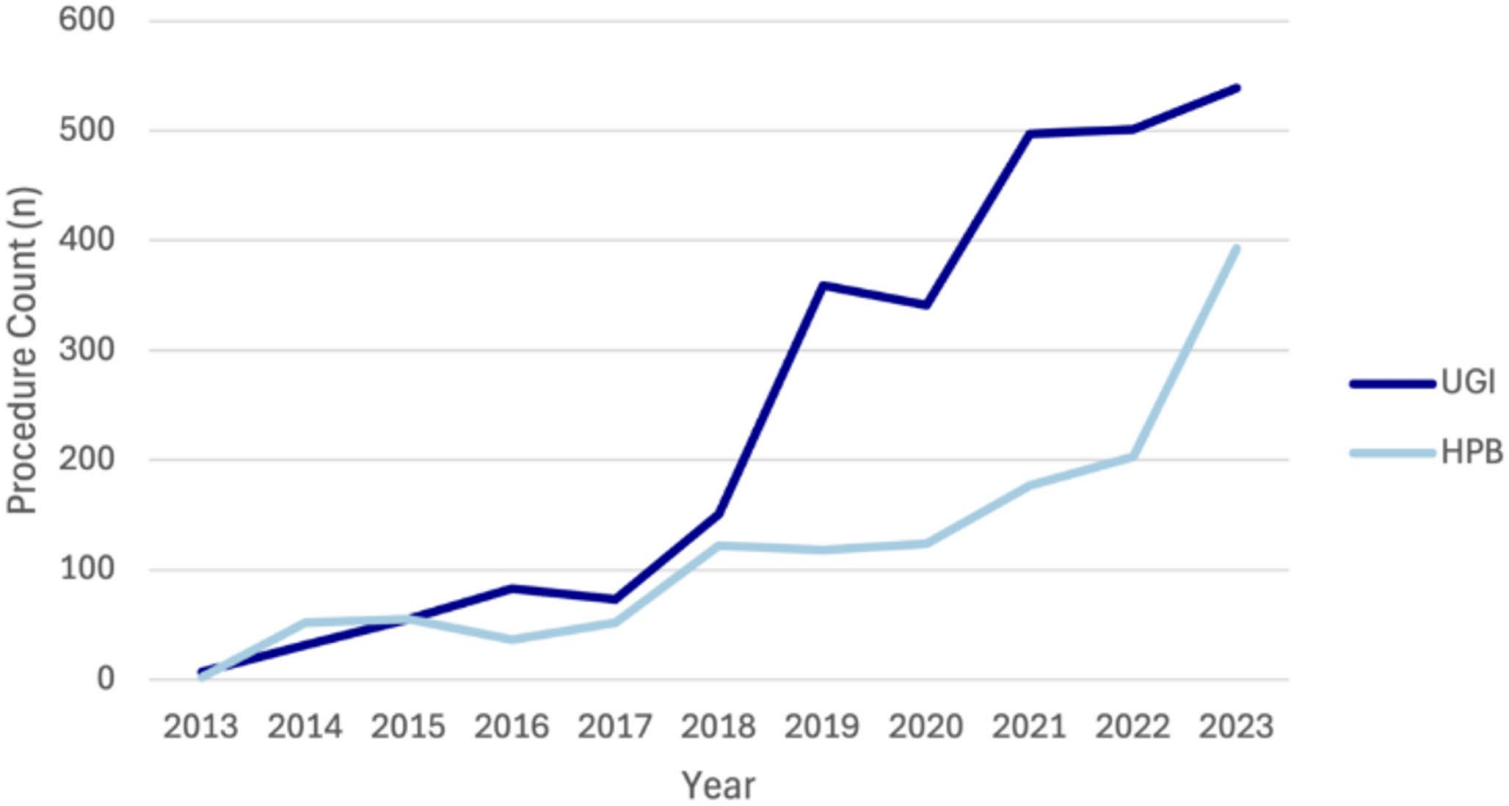
Trends in Robotic UGI and HPB surgery in Australia from 2013 to 2023. Data obtained from Device Technologies

**Fig. 2:**
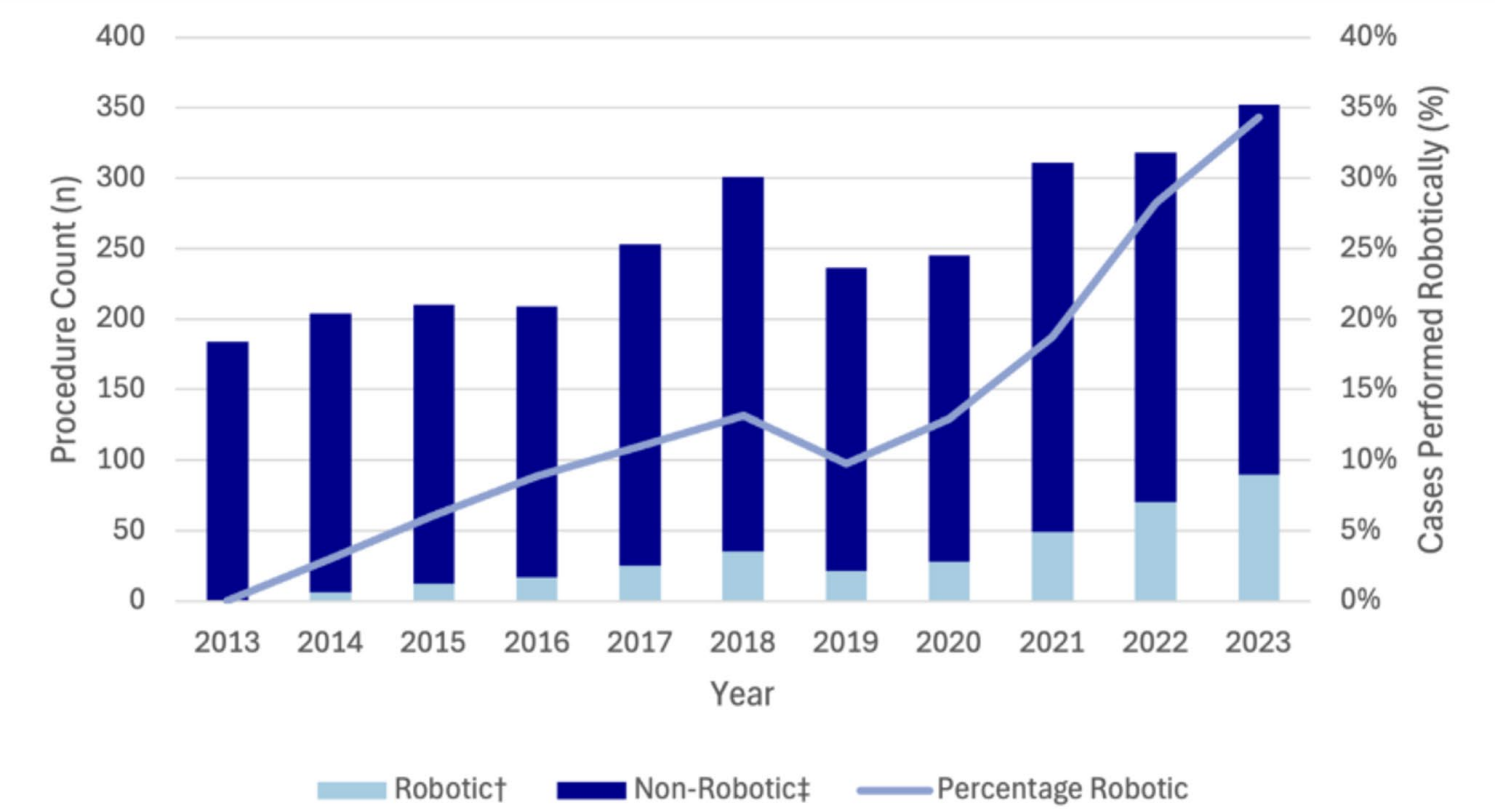
Trends in robotic and non-robotic pancreatectomy in Australia from 2013 to 2023. †Data obtained from Device Technologies. ‡Based on MBS code 30,583 and 30,792

**Fig. 3:**
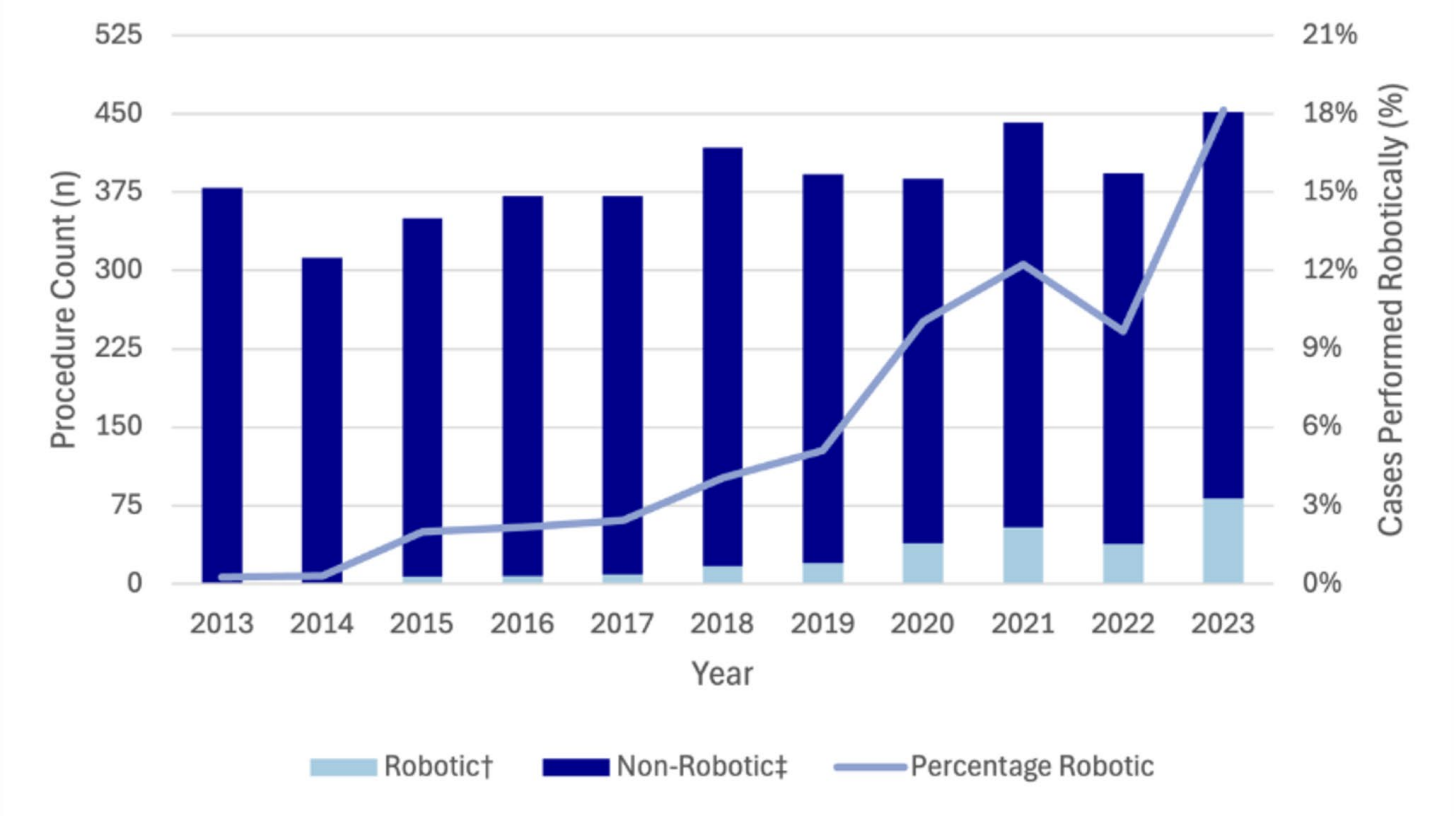
Trends in robotic and non-robotic pancreatoduodenectomy in Australia from 2013 to 2023. †Data obtained from Device Technologies. ‡Based on MBS code 30,584

Descriptive statistics were used to report the frequency and proportion of each robotic procedure by year and category. Procedure-specific graphs were created in Microsoft Excel (Microsoft Corporation, Redmond, WA). To assess overall trends in robotic case volume, a Poisson regression model was used to estimate the overall annual change in UGI and HPB case counts. Year is the independent variable and number of procedures is the dependent variable. Results are reported as an average annual percentage change, with 95% confidence intervals. To assess trends in the proportion of MBS-claimed procedures performed robotically, separate linear regression models were fitted for UGI and HPB procedures with year as the independent variable and robotic proportion, calculated using MBS-claimed procedure counts, as the dependent variable. The choice of linear models rather than logistic was determined by visual inspection of Figs. 1, 2 and 3. Coefficients from these models are reported as absolute percentage-point change per year with 95% confidence intervals. Analysis was undertaken in RStudio (R Core Team, Vienna, Austria).

### Ethics

The study was reviewed by the institutional review board at Adventist Health Care Limited (AHCL). Site authorisation was received on 7 August 2024 (AHCL Reference ID:2024-016).

## Results

Over the study period, 19,663 robotic general surgery procedures were performed, of which robotic UGI surgery made up 13.4% (*n* = 2,637) and robotic HPB surgery made up 6.8% (*n* = 1,334). Figure 1 shows the trends in robotic UGI and HPB surgery over time. Year-on-year, there was an average 33% (95% CI 31%, 35%) increase in the number of robotic procedures. When robotic cases were compared to MBS data as a percentage of MBS-claimed procedures, the proportion performed robotically increased consistently with a year-on-year average increase of 0.16% (95% CI 0.12, 0.20) for UGI procedures and 0.11% (95% CI 0.07, 0.15) for HPB procedures.

**Fig. 4:**
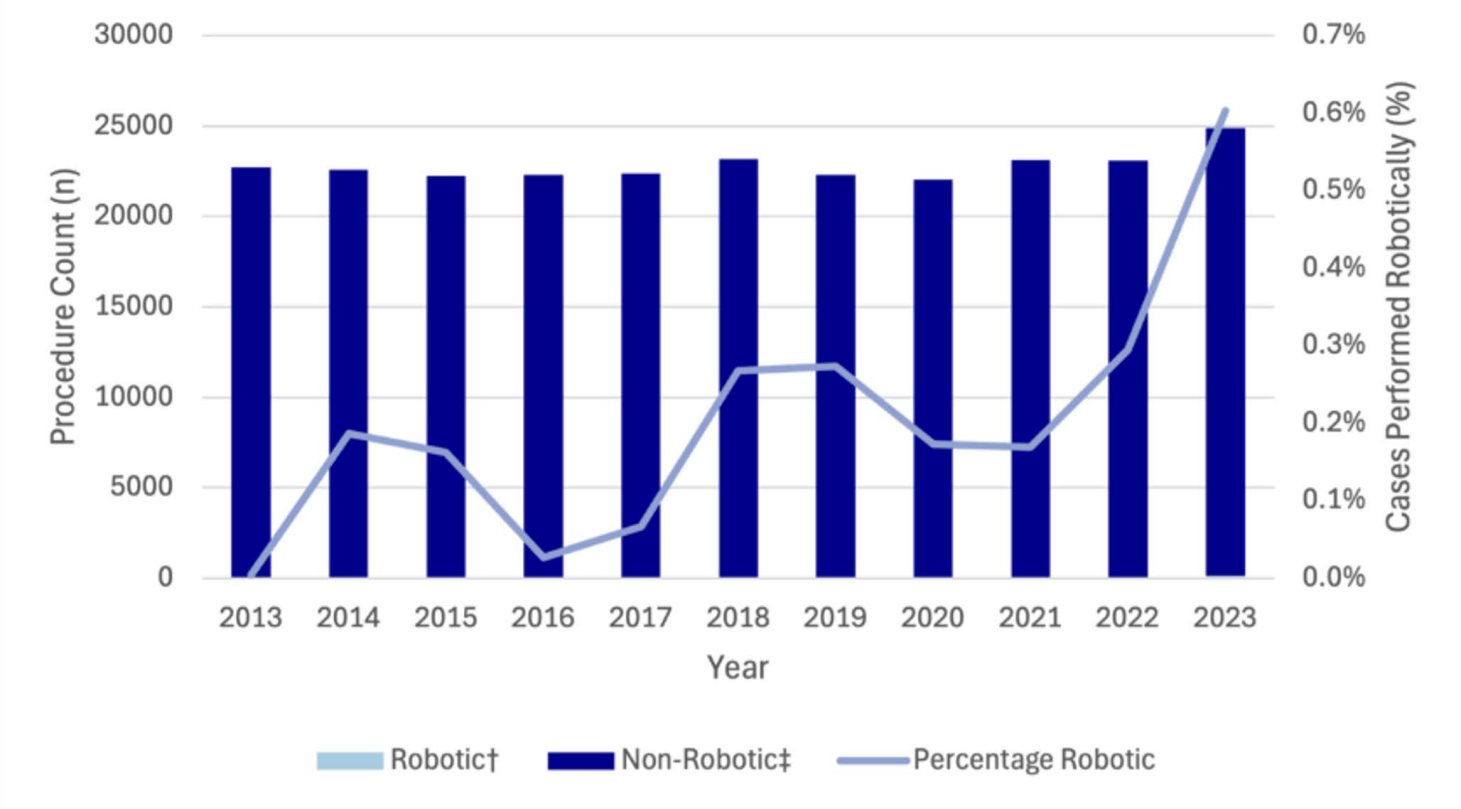
Trends in robotic and non-robotic cholecystectomy in Australia from 2013 to 2023. †Data obtained from Device Technologies. ‡Based on MBS code 30,443, 30,445, 30,448, 30,449, and 30,455

Compared to MBS-claimed procedure volumes, robotic left-sided pancreatectomy increased from 3.0% (*n* = 6) in 2014 to 34.4% (*n* = 90) in 2023 (Fig. 2). Similarly, robotic pancreaticoduodenectomy rose from 0.3% (*n* = 1) of MBS-claimed procedures in 2014 to 18.1% (*n* = 82) in 2023 (Fig. 3). Robotic liver resection also demonstrated growth, increasing from 2.1% (*n* = 29) of MBS-claimed cases in 2021 to 5.4% (*n* = 69) in 2023. Robotic liver resection also demonstrated growth, increasing from 29 procedures in 2021 to 69 procedures in 2023, representing 5.4% of MBS-claimed procedures. Cholecystectomy was the most commonly performed robotic HPB procedure with 150 procedures performed in 2023. While the use of robotic cholecystectomy remained relatively stable from 2013 to 2021 (ranging from 1 to 62 cases annually), a marked increase was observed from 2021, with annual case numbers increasing by 3.8-fold (*n* = 111) between 2021 and 2023. Despite this, the proportion of MBS-claimed cholecystectomies performed robotically in 2023 remained low at 0.6% (Fig. 4).

**Fig. 5:**
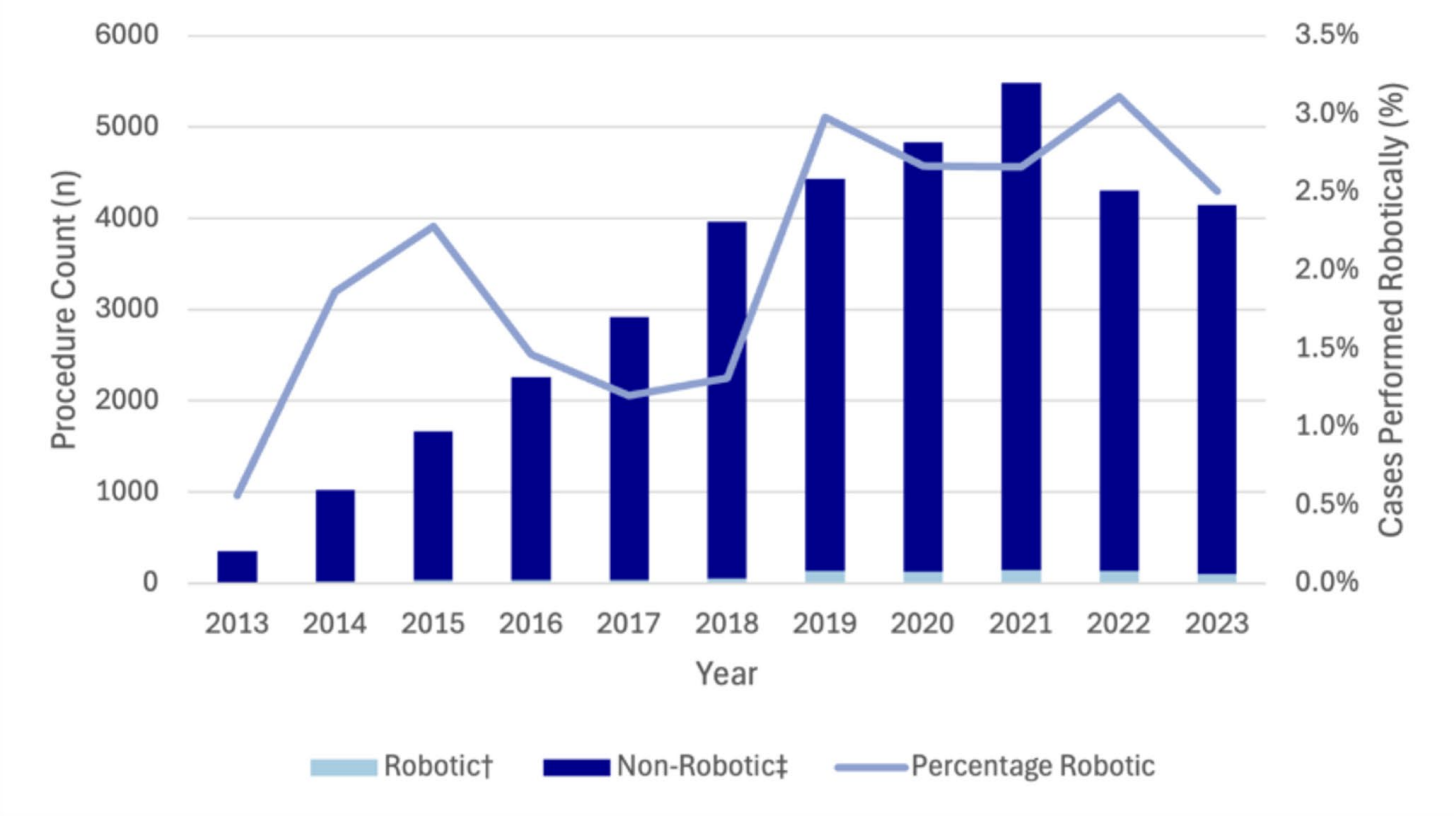
Trends in robotic and non-robotic gastric bypass surgery in Australia from 2013 to 2023. †Data obtained from Device Technologies. ‡Based on MBS code 31,571

**Fig. 6:**
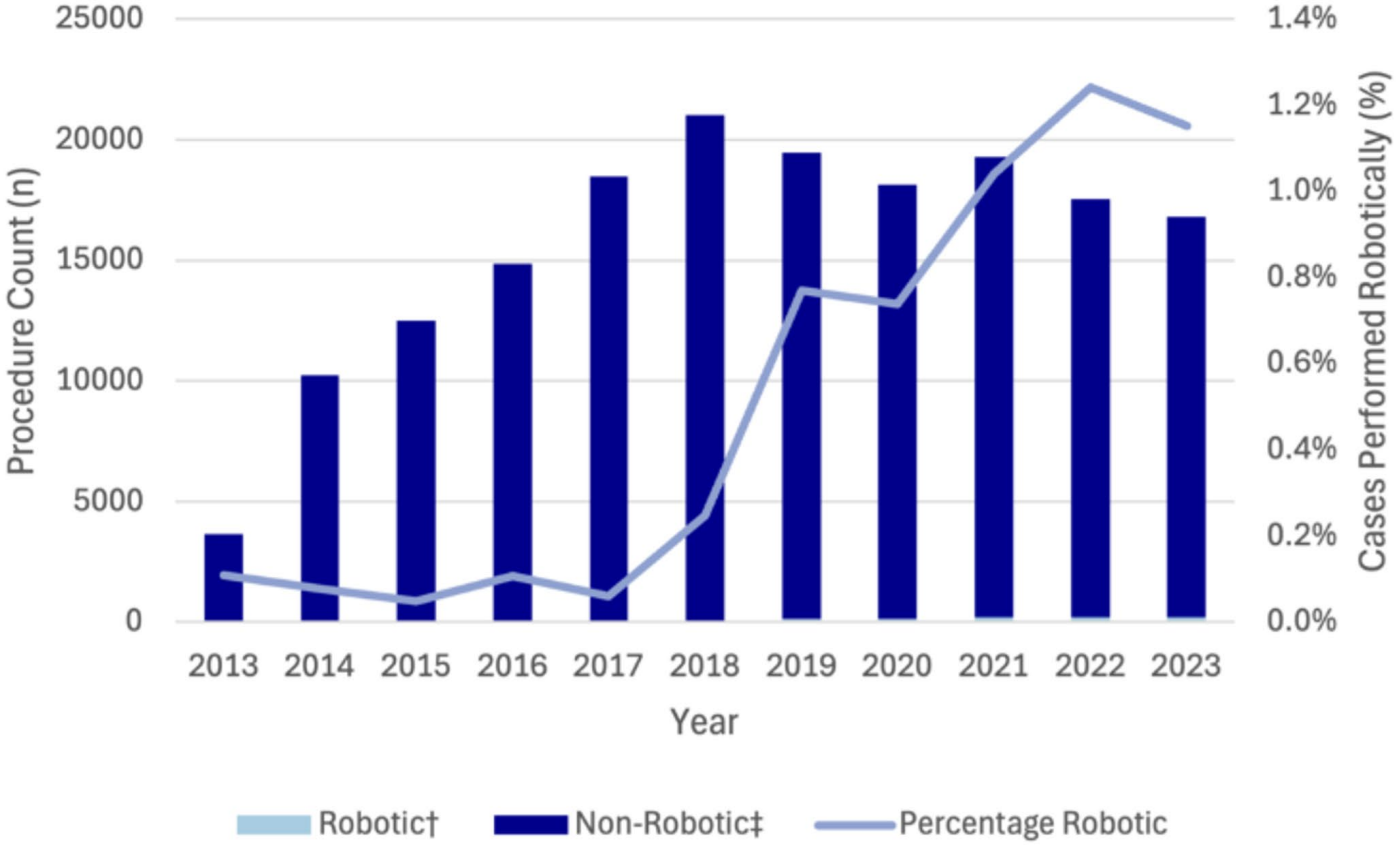
Trends in robotic and non-robotic sleeve gastrectomy surgery in Australia from 2013 to 2023. †Data obtained from Device Technologies. ‡Based on MBS code 31,575

Robotic gastric bypass and sleeve gastrectomy were the most commonly performed UGI robotic procedures in 2023, with 194 and 104 procedures, respectively. Between 2013 and 2022, utilisation of both robotic and non-robotic bariatric surgery increased before declining from 2022 to 2023 by 15.3% (*n* = 54) and 25.8% (*n* = 833), respectively (Figs. 5, 6).

Robotic oesophagectomy was introduced in Australia in 2021, with 66 robotic procedures being performed between 2021 and 2023. Transthoracic approach with chest anastomosis was the most commonly performed oesophagectomy procedure, accounting for 69.7% of all cases (*n* = 46). Since its introduction, the proportion of MBS-claimed oesophagectomies performed robotically has remained stable, making up 10.8% to 11.5% of procedures.

Robotic approaches for other UGI procedures, such as OGM, gastrectomy and hiatal hernia repair have increased over the study period. Robotic OGM has seen rapid adoption since being introduced in 2019, with 6.9% (*n* = 14) of MBS-claimed myotomies utilising a robotic approach in 2023. Similarly, robotic hiatal hernia repair has seen accelerated growth with a 73.9% increase between 2022 (*n* = 46) and 2023 (*n* = 80).

Data for the analyses are provided in the supplementary material.

## Discussion

Using a peer-reviewed method [14], this study provides an initial descriptive analysis of robotic UGI and HPB uptake in Australia using da Vinci robotic data and MBS item data. From 2013 to 2023, the proportion of MBS-claimed procedures performed robotically has increased across all procedure categories, with the most rapid growth observed in complex non-bariatric operations, particularly pancreatectomy and pancreaticoduodenectomy. Although these findings do not completely characterise national trends, they suggest that robotic surgery is becoming increasingly established in Australian UGI and HPB practice.

A notable finding is that we observed a marked rise in robotic pancreatic surgery. In 2023, robotic procedures accounted for one-third of left-sided MBS-claimed pancreatectomies and almost one in five pancreaticoduodenectomies. These findings are consistent with international reports describing increasing use of robotic approaches for pancreatic surgery. For instance, in the US, rates of robotic pancreaticoduodenectomy increased from 0.9% to 8.4% from 2010 to 2020 [20]. These patterns may reflect growing evidence supporting the use of robotic approaches for minimally invasive pancreatic surgery. Laparoscopic pancreaticoduodenectomy has seen limited adoption, largely due to technical difficulty, a long learning curve and the high case volumes required to achieve safety and proficiency [21]. In contrast, robotic surgery can lower some of these barriers, offering improved dexterity and visualisation. Evidence from the EUROPA trial suggests comparable outcomes between robotic and open pancreaticoduodenectomy, although higher rates of pancreas-specific complications have been reported [22]. Robotic distal pancreatectomy also appears to provide advantages over laparoscopic and open techniques in reducing the risk of unplanned splenectomy [23–25]. Collectively, these trends suggest increasing uptake of robotic techniques in complex pancreatic surgery, potentially reflecting growing confidence in the approach.

Compared with pancreatic surgery, uptake of robotic UGI procedures appeared more modest. Although robotic oesophagectomy, gastrectomy, hiatal hernia repair and oesophagogastric myotomy all increased over the study period, absolute case volumes remained relatively low. In contrast, international trends demonstrate increasing adoption of robotic UGI surgery. In the United Kingdom, a survey by the Association of Upper Gastrointestinal Surgeons reported that 61% of UGI resection centres had access to robotic platforms [26]. Similarly, in the United States, the use of robotic techniques for oesophagectomy and gastrectomy has increased substantially over the past decade [27, 28]. Several studies have reported potential benefits in oncological robotic UGI surgery including improved lymph node yield and margin clearance, and reduced hospital stay [29, 30]. In addition, long-term follow-up of the ROBOT trial for robot-assisted oesophagectomy found comparable survival and recurrence rates [31], while a phase II randomised trial for robotic distal gastrectomy demonstrated superior recurrence and survival rates compared to laparoscopic surgery [32]. In contrast to these findings, large registry analyses have raised concerns that early adoption may be associated with higher rates of anastomotic leak and reoperation [28]. Overall, our findings suggest that robotic UGI surgery in Australia remains an evolving area of practice currently limited to a few centres.

In contrast to the overall trends in robotic surgery, the rates of both non-robotic and robotic bariatric surgery plateaued between 2018 and 2023. This may reflect broader changes in obesity management rather than robotic-specific factors. One possible explanation is changing referral patterns associated with increased use of pharmacological weight-loss therapies such as GLP-1 receptor agonists as the preferred treatment for obesity in specific cases [33].

Robotic cholecystectomy emerged as the most frequently performed robotic HPB procedure in absolute numbers despite representing less than 1% of MBS-claimed cholecystectomies performed. Its popularity may reflect its use as a lower-complexity training procedure for developing familiarity prior to more advanced surgery [34–36]. However, recent registry data from 737,908 patients undergoing robotic cholecystectomies in the US demonstrates an associated increased risk of bile duct injury compared to laparoscopic surgery [37]. If robotic cholecystectomy is used as an early training case to build platform familiarity, this should occur within a structured curriculum with explicit strategies to mitigate procedural risk. Similarly, expanded use of other lower-complexity robotic procedures such as hernia repair may also increase individual surgeons’ robotic volume and contribute to system-level competency.

Although robotic UGI and HPB case volume increased steadily over the study period, wider adoption in Australia is likely constrained by limited platform availability. Robotic surgery nationally remains concentrated in high-volume specialties such as urology, colorectal surgery, and gynaecology, which dominate existing robotic programs [3]. As of May 2023, there were 162 robotic platforms across Australia and Aotearoa New Zealand, with only 26 located in public hospitals [3]. UGI and HPB surgery must compete for access to robotic platforms, limiting opportunities for program expansion and case experience. This is especially relevant for rarer and more technically complex procedures such as pancreatoduodenectomy and distal pancreatectomy, which require large case volumes to become proficient [38]. Further growth in robotic UGI and HPB surgery will likely depend on expansion of training pathways and access to robotic platforms at high-volume centres.

Further expansion of UGI and HPB procedures in Australia also highlights the need to consider the economic viability of this technology. While robotic surgeries have higher upfront capital costs, high procedure volumes and optimised theatre utilisation can lower the cost per case, reducing the cost disparity between laparoscopic and robotic procedures [39, 40]. Moreover, in accordance with the Royal Australasian College of Surgeons Robotic Surgery working group statement, higher costs are justified with demonstrable benefits for patients and the healthcare system [3]. Further cost-effectiveness evaluations that incorporate downstream outcomes such as long-term clinical outcomes are needed to inform public-sector investment.

This study has several limitations. Firstly, robotic case counts and comparator procedure volumes were derived from different sources and may not represent the same underlying procedural population. Secondly, MBS data does not capture procedures performed in public hospitals or outside the Medicare system. As robotic platforms are concentrated in the private sector [3], data presented as a proportion of MBS-claimed procedures are more representative of private-sector practice than national practice as a whole. Thirdly, the robotic procedure dataset does not include surgical outcomes, including whether procedures were completed as robotic cases or required conversion to open surgery. Lastly, although the da Vinci Robotic System remains the predominant platform for general surgery in Australia, other systems have been introduced since 2019. Procedures performed on these platforms were not captured, potentially leading to an underestimation of the total robotic case volume.

## Conclusion

Robotic surgery for UGI and HPB procedures has increased between 2013 and 2023, with a recent shift towards complex non-bariatric robotic surgery. Furthermore, the proportion of MBS-claimed UGI and HPB procedures performed robotically increased over the study period. These trends highlight the need for the development of national outcome registries, training programs and practice guidelines in robotic surgery. Additionally, future studies should consider the cost-effectiveness and feasibility of public-sector use.

## Electronic Supplementary Material

Below is the link to the electronic supplementary material.

Supplementary Material 1

## Data Availability

Procedure counts were extracted from the Medicare Benefits Schedule (MBS) Item Reports website using specific MBS codesData on the number of general surgery robotic procedures was obtained from Device Technologies Australia, the local distributor of the Da Vinci Robotic System (Intuitive Surgical, Sunnyvale, CA, USA).
